# The Role of School Connectedness and Friend Contact in Adolescent Loneliness, and Implications for Physical Health

**DOI:** 10.1007/s10578-022-01449-x

**Published:** 2022-10-19

**Authors:** Yixuan Zheng, Margarita Panayiotou, Dorothy Currie, Keming Yang, Charlotte Bagnall, Pamela Qualter, Joanna Inchley

**Affiliations:** 1 Manchester Institute of Education, University of Manchester, Oxford Road, Manchester, M13 9PL UK; 2 School of Medicine, University of St Andrews, St Andrews, UK; 3 Department of Sociology, Durham University, Durham, UK; 4 MRC/CSO Social and Public Health Sciences Unit, University of Glasgow, Glasgow, UK

**Keywords:** Loneliness, Adolescents, School connectedness, Peer contact, Friendship, Physical health

## Abstract

The current study investigated how adolescents’ loneliness relates to school connectedness, classmate support, teacher support, and offline and online communication with friends. We also examined the association between loneliness, physical health, and sleep. Data came from the Scottish Health Behavior in School-aged Children (HBSC). The total sample was 2983 adolescents (F = 1479 [49.6%]) aged 14–17 years (*M* = 15.66, *SD* = 0.39) from 117 secondary schools in Scotland. Results showed that (1) higher teacher support, classmate support, and offline contact with friends predicted lower levels of loneliness, (2) online friendship engagement predicted higher levels of loneliness, and (3) poor health and sleep were positively associated with loneliness. The study offers new findings, highlighting the role played by classmates/peers and teachers in reducing loneliness. Supporting previous research, we also found associations between loneliness, poor sleep, and worse physical health.

**Supplementary Information:**

The online version contains supplementary material available at 10.1007/s10578-022-01449-x.

## Introduction

Loneliness is a common troublesome emotional state [1–4], caused by the inconsistency between ideal and actual interpersonal relationships [5]. There is a growing body of literature showing the negative impacts of loneliness for young people, including poorer academic performance [6], and worse mental and physical health [7, 8]. Further, when loneliness continues from adolescence to young adulthood, it often contributes to adverse effects on educational qualifications, mental health, career prospects, and life satisfaction [9, 10]. Importantly, Twenge et al. [11] found an increase in the prevalence of adolescent loneliness worldwide, highlighting the urgent need to identify effective strategies to reduce loneliness during early adolescence.

Previous studies have shown three essential interpersonal relationships throughout adolescence: with family members, teachers, and peers [12, 13]. Relationships with family members, especially parents, are particularly significant in relation to children’s mental health [14]. But, when children begin school, teachers also become important in a child’s life [15], supporting students’ emotional functioning and academic outcomes [16]. During the adolescent years, young people develop increasing autonomy and establish stronger relationships with peers [17, 18], making peers important sources of support [4]. While family relationships remain essential during this time, peer relationships become increasingly important in predicting well-being [19, 20]. Indeed, supportive relationships with classmates predict reduced internalizing problems [21–23], through the development of new friendships [24], but also through the general classroom climate of peer support [25, 26]. Close relationships with friends, one of the supportive peer relationships, are positively associated with emotional adjustment during adolescence [27]. Among young people reporting loneliness, Spithoven et al. [28] identified a significant aspiration for engaging with reliable friends. Taken together, empirical findings have showed the importance of positive interpersonal support for adolescent emotional well-being. In the present study, the contribution of these social factors to loneliness is explored in the contexts of school connectedness and friend contact.

Evidence suggests that school is the most dominant setting for adolescents to experience loneliness [29, 30], with peer relationships being the major source of loneliness. Students reported that support from teachers in relation to loneliness was limited, with their loneliness being trivialized [30]. Despite such evidence, there are few studies concerned with the school social context, with discussions remaining narrow in focus; most studies dealt only with a limited perspective within the school environment, such as the teacher’s role [13, 31], or simply involved the school setting as one aspect within multiple social contexts [32, 33]. In the current study, using data from the Scottish Health Behavior in School-Aged Children (HBSC) Survey, we address this gap by exploring whether school connectedness, in the form of classmate and teacher support, influences reports of loneliness among young adolescents. Moreover, we explore online communication with friends and their offline (face-to-face) contact, exploring how differential contact with close friends is linked to loneliness. De Looze et al. [34] found that adolescents who reported daily online communication with friends spent more time with their friends offline, suggesting that online contact with friends may be associated with lower rather than higher levels of loneliness. Thus, the current paper explores whether the time spent with friends on- and off-line impacts loneliness among adolescents.

Secondary data analysis also provided us with an opportunity to explore the relationship between loneliness and poor health, enabling an examination of health and sleep. Loneliness among adults is associated with poorer sleep and poor health (for review, see [35]), but there is only one study among young adolescents using data from a large population study [36]. That population study showed that loneliness was associated with poorer sleep and poor self-reported health among Danish adolescents. Thus, exploration of that association using data from another population study is essential to explore whether the relationships are consistent across samples.

## Loneliness and School Connectedness

It has been argued that the school is a primary context for young people to experience loneliness [33, 37], given the amount of time they spend there, in the company of other people. Indeed, next to family connectedness, school connectedness has been recognized as second in importance as a factor associated with students’ psychological problems [38]. Extant literature showed that experiences of school connectedness are positively correlated with students’ mental health and well-being [39–41]. Further, students who report higher levels of school connectedness are also more likely to achieve academic success [42–44]. However, to date, there have been limited attempts to explore the association between feelings of school connectedness and loneliness. Such exploration is essential if we are to understand how loneliness develops among young people, how it is maintained, and how it’s effects might be mitigated.

School connectedness is defined as the sense of attachment and commitment a student feels in school [45], and includes the social relationships students have with their teachers and peer groups, and the support they provide. School connectedness is an important dimension of the school climate [46], and it is specifically related to mental health [47]. Using reports of teacher and classmate support, the current study explores the role of school connectedness in relation to adolescents’ loneliness.

## Loneliness and Friend Contact

When considering social factors that might contribute to loneliness, another important factor is interaction with friends. Providing social and emotional support, high-quality friendships are critical for the general well-being of young people [48, 49]. In contrast, poor friendship experiences negatively contribute to students’ feelings of loneliness [50–52].

Online Communication has become an integral part of the after-school schedule, providing young people with opportunities to build virtual social networks, which could contribute to the maintenance of friendships [53, 54]. In addition, communication in cyberspace could help facilitate friendship building for young people who may face social difficulties [55–58]. However, recent cross-sectional evidence found excessive use of social media is associated with higher levels of psychological distress and loneliness [33, 59]. In a recent review of current evidence, Smith et al. [60] showed that use of social media technology can potentially be both a barrier and a facilitator of personal well-being for young people. It is possible that electronic media use leads to fewer face-to-face interactions with friends, which may lead to fewer open discussions that are important to friendships, and also less engagement with real-world friends as one makes new social relationships online. Nevertheless, it is also possible that adolescents who are active users of social media spend more face-to-face time with friends, because cyberspace encourages communication with existing friends, with adolescents using electronic media to communicate about where and when to meet and what to do offline. The current paper makes an important contribution to this debate by exploring whether online and offline peer contact with friends is linked to loneliness during adolescence.

## Loneliness and Poor Health

Previous research with adults (for review, see [35]) has shown loneliness is associated with a poor night’s sleep and poor health, and there are several studies linking loneliness and poor health among young people [36, 61]. Compared to non-lonely adolescents, youth who report loneliness are more likely to suffer from physical health problems, with higher levels of subjective health complaints [62], more health-compromising behaviors (i.e., heavy smoking, excessive alcohol use, drug use) [3], higher frequencies of doctors’ visits [63], and the greater chronic disease risks (i.e. respiratory disease, cardiovascular disease) in early adulthood [7, 64]. Furthermore, lonely adolescents may be more often affected by sleep disturbance [36]. Noteworthy, several studies have shown that physical health and sleep problems can be linked with poorer school performance [65, 66], which may contribute to lower academic grades and general engagement in school. However, how those are affected when other important variables such as school social experiences are taken into account is not known. In addition, we found only one study using data from a large population-based study [36], and there is a need to explore the association between loneliness and health using other data from population studies with youth.

## The Current Study

Using data from the Scottish Health Behavior in School-aged Children (HBSC), the overall aim of present research is to explore the role of school connectedness and friend contact in relation to loneliness, and to explore the associations between loneliness, physical health, and sleep among young people. The present research aims to offer important insights into social variables that might play a significant role in young adolescents’ loneliness using data from a large representative population study; they include classmate support, teacher support, offline (face-to-face) contact with friends, and online communication with friends. Centered on loneliness, our research questions follow a conceptual model: social variables → loneliness → poor health and sleep. We have three research questions as follows: (1) Are higher levels of support from teachers and classmates associated with loneliness, (2) Is the frequency of contact with friends on- and off-line associated with loneliness, and (3) do adolescents who report higher loneliness experience more health problems.

## Method

### Design and Participants

The current study used data from the 2013/2014 Health Behavior in School-Aged Children (HBSC) survey from Scotland, funded by NHS Health Scotland. The data were part of the larger cross-national HBSC study conducted every four years in collaboration with the World Health Organization Regional Office for Europe [67]. The main focus of the HBSC study is the physical and mental health of young people aged 11, 13 and 15 years in family, school and peer contexts, which underlines the interplay between individual and social settings. A social psychological approach is also adapted, highlighting the role of psychological factors in explaining individual health behaviors. The Scottish HBSC survey followed the international HBSC survey protocol [68]. Using standardized and validated questionnaires, the cross-sectional survey collected the following data: demographic factors (e.g., age, gender); social context (e.g., family life, peer relations, school environment); health behaviors (e.g., eating habits, physical activity and weight control behavior); risk behaviors (e.g., substance use, sexual health, bullying and fighting); health outcomes (e.g., self-rated health, health complaints, body image); and well-being (e.g., life satisfaction, self-confidence, feeling left-out and feeling lonely). Detailed information regarding the study can be accessed at www.hbsc.org.

For the current study, data from the 2013/2014 cohort for the HBSC survey in School were used. The sample for the current study comprised 2983 children (F = 1479 [49.6%]) ages 14–17 years (*M* = 15.66, *SD* = 0.39) from 117 secondary schools in Scotland. The sample was designed to be nationally representative using the school class as the primary sampling unit, stratified by Local Education Authority and type of funding (public or private). Ethical approval was granted by the University of St Andrews School of Medicine Ethics Committee. All participants were informed about the study in advance and given the opportunity to withdraw. Data were collected through anonymous self-report questionnaires administered by teachers in the classroom setting.

### Measures

We constructed a list of possible correlates of loneliness based on the evidence in the existing literature, and then explored the HBSC survey to determine which manifest variables would be suitable to create latent variables of each construct of interest. We used confirmatory factor analysis (CFA) to confirm the structure and validity of each latent construct, determining whether the manifest variables could be appropriately used. The results of the CFA can be found in Supplementary material 1.

#### Loneliness

A latent variable of loneliness was created using the following items: “Last week, did you feel lonely”, “how often do you feel left out of things”, and “how often do you feel close to others”. Participants used a five-point Likert scale that ranged from “never” (0) to “always” (4). The third item was reverse scored so that higher scores indicate higher loneliness.

#### Teacher and Classmate Support

Adolescents were asked the extent to which their teachers care, accept, and offer additional support to students (e.g., “I feel like my teachers care about me as a person”). Classmate support was measured by three items assessing the extent to which students feel good together, help, and accept each other (e.g., “most of my classmates are kind and helpful”). Participants rated each item using a 5-point scale from “strongly agree” (0) to “strongly disagree” (4). Initially, then, higher scores on each item indicated lower classmate or teacher support, but we reverse coded for ease of interpretation in our analyses so that higher scores reflected higher support. In our analyses, we created two latent variables using the items, one that reflected teacher support and the other that indicated classmate support.

#### Offline Contact with Friends

A latent variable was created that measured the extent of face-to-face contact with friends. Adolescents were asked “How often do you meet your friends outside school time…? before 8 pm?/after 8 pm?” Response options included “Hardly ever or never” (1), “Less than weekly” (2), “Weekly/Daily” (3). The latent variable also included the items “easy to talk to best friend”, “talk to friends of the same sex”, and “talk to friends of the opposite sex”; response options ranged from “very easy” (0) to “I do not have this person/I do not do this” (4), and those were reverse coded for analyses, such that higher scores represented more contact with friends.

#### Online Communication with Friends

This was measured using the item: “How often do you…? Talk to your friends on the phone or internet-based programs such as FaceTime or Skype; Contact your friends using texting/SMS; Actively contact your friends using instant messaging (e.g., BBM, Facebook chat); Contact your friends using other social media, such as Facebook (posting on wall, not chat), Myspace, Twitter, Apps (e.g., Instagram), games (e.g., Xbox), YouTube, etc.” Response options were “hardly ever or never” (0), “less than weekly” (1), “Weekly” (2), and “Everyday” (3).

#### Health

The HBSC Symptom Checklist (HBSC-SCL) was used to measure adolescents’ subjective health complaints. The HBSC-SCL asks youth to indicate how frequently they experienced health symptoms in the last 6 months. The eight health complaints are headaches, stomach-aches, backache, feeling low, bad temper, nervousness, sleep difficulties, and dizziness. Adolescents were asked how often they experienced these symptoms over the last 6 months, with five response options (“about every day”, “more than once a week”, “about every week”, “about every month”, and “rarely or never”). We reverse coded these items so that higher scores represented *poor health*. Previous research has shown support for a two-factor solution for the HBSC-SCL: a dimension of psychological health complaints (feeling low, irritability or in a bad mood, feeling nervous and sleeping difficulties) and a dimension of somatic health complaints (headache, stomach-ache, backache, and dizziness) [69, 70]. We did not include psychological complaints in our analyses because they map too closely with loneliness according to research [4]; instead, we explored “health complaints” using participants’ reports of headaches, stomach-ache, backache, and sleep difficulties only. Our factor analyses supported a single factor solution, with only “sleep difficulties” having a poor factor loading. Thus, in our analyses, we treated “health complaints” as a single latent factor consisting of somatic health complaints (headache, stomach-ache, and backache) and “sleep difficulties” as a manifest variable in our analyses.

### Analysis Plan

Missing data on items ranged between 0.7 and 6.4%. All available data were used in the analysis with pairwise present under the assumption of missing at random. The constructs of the hypothetical model were modelled as latent variables because that estimates and removes item-level measurement error, providing more accurate estimates of model pathways [71]. Models were estimated in Mplus 8.2 using weighted least squares means and variance adjusted (WLSMV) estimation, which can handle models with large sample sizes and many latent factors [72]. Several indices were used to assess model fit as follows: the Tucker-Lewis index (TLI), Comparative fit index (CFI), root mean square error of approximation (RMSEA; including 90% Confidence intervals), and standardized root mean squared residual (SRMR). Models with TLI and CFI values above 0.95, RMSEA values below 0.06, and SRMR values below 0.08 were considered to have good fit [73]. The residual correlation matrix was also assessed to identify the levels of model misfit [i.e., correlations > 0.10; 74]. Given that students were nested within schools (*N* = 117, mean cluster size = 25.4), the goodness-fit-statistics and the standard errors of the parameter estimates were adjusted to account for the dependency in the data (using Type = Complex in Mplus).

#### Measurement models

Confirmatory factor analysis (CFA) was conducted first in order to confirm the structure and validity of each latent construct, followed by the estimation of the full measurement model—where all variables are freely correlated—as a viable model must be established prior to evaluating the structural model [72]. Supplementary material 1 provides details of the measurement models. The internal consistency of the constructs was assessed through unidimensional (ω) composite reliability (known as McDonald’s omega) as proposed by Raykov and Marcoulides [75], which provides more accurate estimates when τ-equivalency is violated.

## Results

Internal consistency coefficients and latent correlations among the study variables are shown in Table 1. As expected, poor health and sleep were negatively associated with social support variables and positively with loneliness. Given that the fit of the saturated models for loneliness, classroom support, teacher support, and offline contact could not be assessed, their loadings were examined instead, which were shown to be substantial (λ = 0.50–0.92). Good model fit was found for health (χ^2^ (2) = 2.65, *p* > 0.05; RMSEA = 0.010 (90% CI [0.000, 0.039]), *p* > 0.05; SRMR = 0.004; CFI = 1.00; TLI = 1.00) and online communication with friends (χ^2^ (2) = 11.7, *p* < 0.01; RMSEA = 0.041 (90% CI [0.020, 0.065]), *p* > 0.05; SRMR = 0.009; CFI = 0.996; TLI = 0.989). Finally, the overall measurement model was excellent (χ^2^ (169) = 963.71, *p* < 0.01; RMSEA = 0.040 (90% CI [0.037, 0.042]), *p* > 0.05; SRMR = 0.036; CFI = 0.973; TLI = 0.966), with low misfit (97.4% of residual correlations < 0.10), and was, therefore, considered adequate for use in subsequent analyses.

**Table 1:** Internal consistency, and latent correlations between the study variables

	1	2	3	4	5	6	7
1. Poor health	–						
2. Poor sleep	0.63***	–					
3. Loneliness	0.55***	0.50***	–				
4. Classroom support	− 0.29***	− 0.27***	− 0.53***	–			
5. Teacher support	− 0.23***	− 0.21***	− 0.41***	0.51***	–		
6. Offline contact with friends	− 0.16***	− 0.14***	− 0.29***	0.30***	0.20***	–	
7. Online communication with friends	0.01	0.01	0.02	0.08**	− 0.08**	0.45***	–
Composite reliability ω [95% CI]	0.74 [0.73, 0.76]	–	0.69 [0.66, 0.71]	0.79 [0.78, 0.81]	0.85 [0.84, 0.86]	0.75 [0.73, 0.78]	0.67 [0.64, 0.69]

CI= confidence interval

**p < 0.01, ***p < 0.001

**Fig. 1:**
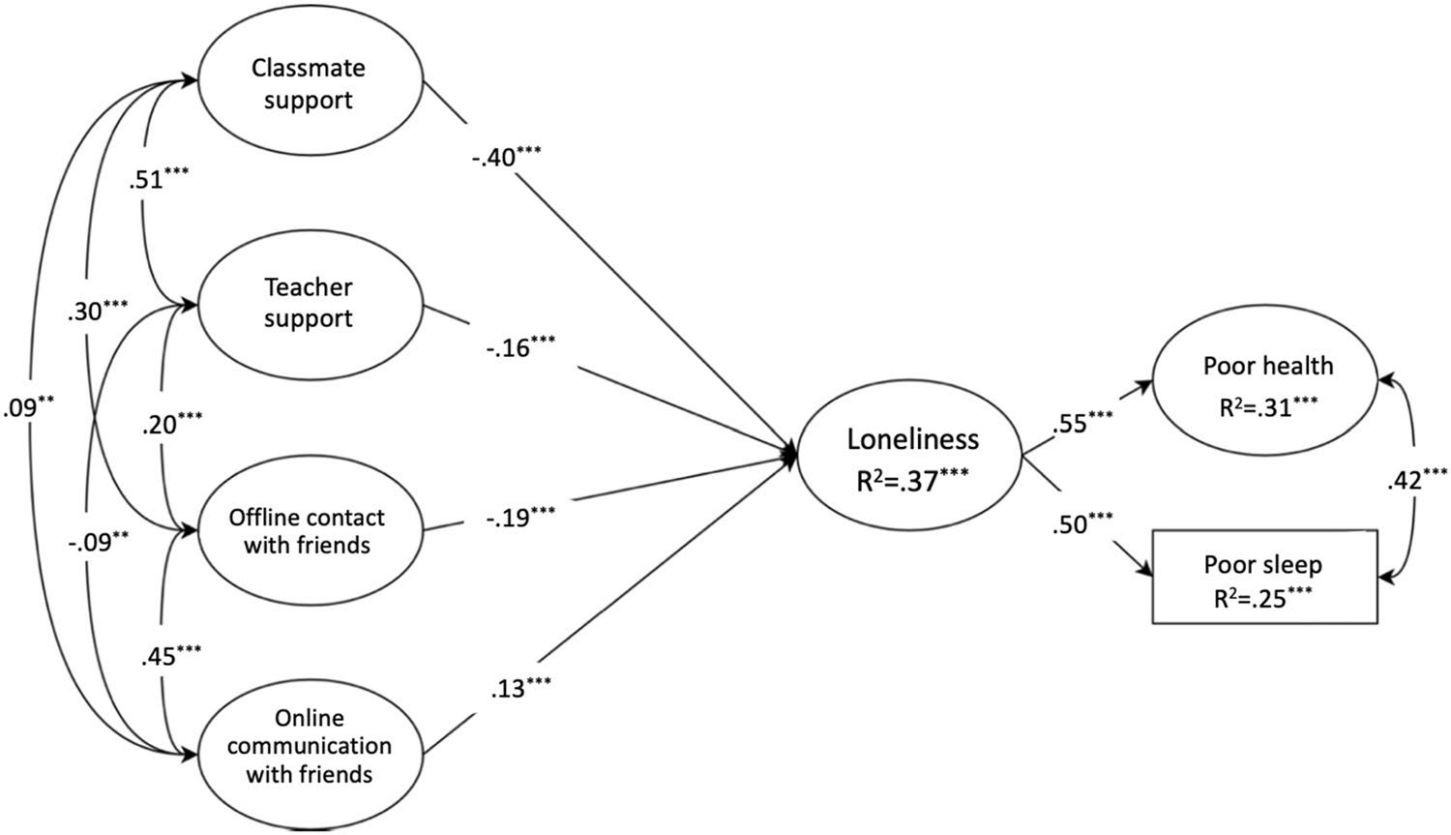
Hypothesized structural equation model

The hypothesized structural model (see Fig. 1) was shown to have a good fit (χ^2^ (177) = 1361.59, *p* < 0.01; RMSEA = 0.047 (90% CI [0.045, 0.050]), *p* > 0.05; SRMR = 0.048; CFI = 0.959; TLI = 0.952). 31% and 25% of the variance in health and sleep were predicted by the overall model. Accounting for the shared variance between the four social variables, these were shown to be significant predictors of loneliness: higher teacher support, classmate support, and face-to-face contact with friends predicted lower levels of loneliness, and more frequent social media use predicted higher levels of loneliness. Loneliness was shown to significantly predict worse health and sleep.

## Discussion

Using data from a large sample of Scottish adolescents from the HBSC survey, we examined the association between loneliness and (1) teacher and classmate support, and friend contact in person and online, and (2) physical health, and sleep among young people. As in previous research, we found that lower levels of offline contact with friends, and lower teacher and classmate support predicted reports of loneliness, suggesting that different types of social relationships impact how lonely adolescents report themselves to feel. In addition, we found that spending more time in online communication with friends predicted higher levels of loneliness, supporting the argument that online friendship engagement may not be an effective substitute for face-to-face contact in combatting loneliness among adolescence. Consistent with previous literature using data from the Danish HBSC survey [36], we found that loneliness significantly predicted worse health and poorer sleep among adolescents ages 14–17 years. Together, our findings support the need for interventions for loneliness to alleviate its negative effect on health and sleep, which have been found previously to impact school attainment and school liking [76, 77]. Our study also identifies modifiable aspects of the school social environment that could reduce loneliness. Combined with other recent evidence that showed school climate, specifically teacher support, teacher interest, peer competition and cooperation, and discrimination were important in understanding student loneliness [6], our findings support the idea that making changes to the school social environment could potentially be effective at reducing the prevalence of loneliness among school-aged adolescents, and is within the reach of most schools and teachers.

### Loneliness and School Connectedness

Our findings indicate that school connectedness is an important protective factor for loneliness during adolescence. We propose that by focusing on increasing support between students and teachers, and students and their classmates, schools may reduce loneliness; a focus on increasing belonging and companionship can ultimately reduce feelings of disconnection. In other recent work [6] that explored loneliness among school-aged adolescents from around the world, supportive school climates that were free from prejudice were associated with lower reports of loneliness from students. Thus, there are important benefits of increasing opportunities to engage positively and supportively with others in the school community that lead to reductions in loneliness. It is noteworthy that teacher support shows a weaker association with pupil loneliness than classmate support. Given findings from other recent work [30], it is likely there is dissonance between lonely students’ expectations and their actual experience of teachers’ emotional support. Teachers may, therefore, find it difficult to offer appropriate support to students in the “eye of the storm”. Previous work has found that most teachers are not well equipped to deal with loneliness from the perspective of students [13], and often find themselves dismissing the negative effects of loneliness because they simply do not know what to do [30]. Compared to teachers, we found that classmates are more important in relation to loneliness in school. Over the adolescent period, peers become increasingly important as individuals establish more independence from adults such as parents, teachers, or school staff [22]. Classmates are seen as one of the most available and helpful sources of support [78]. Thus, support from the general classmate group, where there is consistent and tangible social acceptance means loneliness levels are low. These findings highlight the importance of prioritizing supportive connections between classmates as a means of relieving student loneliness.

### Loneliness and Friend Contact

While classmate support was a stronger predictor of loneliness than peer contact, we found that frequent contact with friends was associated with lower loneliness. Based on previous studies (for review, see [52]), it is likely that face-to-face contact enables young people to build confidence in their social skills and develop good quality relationships. Building on previous research [79], our findings suggest that face-to-face interaction provides differential benefits for young people. When combined with our findings about online communication, that showed higher contact with friends on social media predicted higher levels of loneliness, it appears that there is something important about face-to-face contact with friends that is not found through online peer interaction.

### Loneliness and Poor Health

We found a positive association between loneliness, poor health, and poor sleep among young adolescents. Corroborating research by Eccles et al. [36], the hypothesized association was strengthened by the present study conducted in another large representative population data, and in a model that also included predictors of loneliness.

Poor health and sleep are considered serious threats to adolescents’ school attendance, learning capacity, and academic achievement [80–82]. Our findings support previous work, show that loneliness puts young people at risk of both poor health and sleep, and suggest that lonely adolescents are significantly disadvantaged. It is likely the relationship between loneliness and poor health and sleep difficulties exposes them to the risk of employment difficulties as adults [10, 83].

### Limitations of the Current Study

Several study limitations need to be considered; recommendations for future research work also need to be proposed. First, it is important to recognize the current study was cross-sectional, and the developmental effects of teacher and classmate support, and face-to-face and online friendship contact remain unclear. Longitudinal research should be conducted to explore the relationship between different types of social relationships and loneliness across the adolescent years. Another potential limitation is that the sample of adolescents were from one country and results may have been influenced by wider social and cultural norms. Indeed, it is known that the behaviors and cognitive abilities of people are influenced by their cultural backgrounds and expectations, especially in school settings [84]. Consequently, young people’s perceptions of supportive relationships, particularly at school, may be different within different culture contexts. Future research should explore the influence of school support and friend contact on loneliness across a variety of cultural contexts. It is also important to recognize the data used were from the 2013/2014 HBSC survey. It is possible that the attitudes towards social media amongst adolescents have changed as the use of social media has become increasingly pervasive. Future studies will want to examine the findings with regards to on- and off-line contact by collecting and analyzing more recent data.

### Implications

Despite the aforementioned limitations, the current study makes a valuable contribution to the literature on how different social relationships influence loneliness during adolescence. Further, the large sample allowed us to explore how loneliness related to physical health and sleep, adding additional data showing the negative impact of loneliness on wider aspects of adolescent’s lives. The current study has important implications for practice, providing ideas for how to reduce loneliness among adolescents. Given the significance of school connectedness to loneliness, we highlight the need for teachers to offer support to their students. Research to date has proposed two types of teacher support that could be important for reducing loneliness among students—instrumental and emotional teacher support [85]. Instrumental support refers to assistance and guidance that teachers could provide for students’ academic achievement and personal development, whereas emotional support refers to students’ perception of the teachers as caring, warm, empathic and trustworthy [86]. We suggest teachers gain adequate knowledge of those different types, be predictive of students’ help-seeking behavior, and combine those two kinds of types more flexibly when offering support. In addition, intervention work needs to consider the role of face-to-face peer support for reducing loneliness. Indeed, in research and practice, the emphasize has been on the significance of friends for a long time, whilst neglecting the role of wider peer groups such as classmates, with whom young people spend a great amount of time at school. To sum up, we recommend, based on our findings, the co-action of classmates and teachers to create healthy and inclusive school climates. We would expect such action to make school a significant setting for interventions that reduce students’ loneliness; based on our findings, we would expect such interventions to have positive consequences for sleep and physical health.

## Summary

The present study explored (1) the role of school variables and friend contact on loneliness, and (2) the associations between loneliness, physical health, and sleep among young adolescents. Our results extend the current literature on young people’s loneliness by showing that loneliness is related to lower levels of teacher support, lower classmate support, and having less face-to-face contact with friends; while communicating with friends on social media appeared to be positively linked to loneliness. Additionally, we found a positive association between loneliness, poor health, and poor sleep during adolescence, supporting the necessity for interventions to relieve loneliness and alleviate its adverse impacts. Notwithstanding its limitations, this study provided valuable insights into ways to reduce loneliness within schools, and highlighted the particular importance of school connectedness and supportive peer/classmate relationships.

### Supplementary Information

Below is the link to the electronic supplementary material.

Supplementary file1 (DOCX 23 kb)

## Data Availability

The HBSC 2013/14 data are available via the UK Data Archive.
