# Supplementary material for: The Role of School Connectedness and Friend Contact in Adolescent Loneliness, and Implications for Physical Health
Source: Child Psychiatry Hum Dev. 2022 Oct 19;55(3):851–60. doi: 10.1007/s10578-022-01449-x (PMC11060972; doi:10.1007/s10578-022-01449-x)
Supplement: Supplementary file 1 — Supplementary file1 (DOCX 23 kb) [file 10578_2022_1449_MOESM1_ESM.docx]

# The Role of School Connectedness and Friend Contact in Adolescent Loneliness, and Implications for Physical Health.

Yixuan Zheng, Margarita Panayiotou, Dorothy Currie, Keming Yang, Charlotte Bagnall, Pamela Qualter, and Joanna Inchley

**Supplementary Material**

| Online Communication with Friends | | |
| --- | --- | --- |
| **Items** | **λ** | **h^2^** |
| Talk to friends on phone/internet | .72 | .52 |
| Using texting/SMS | .67 | .41 |
| Using instant messaging | .78 | .61 |
| Other social media | .68 | .46 |
| **Model fit** |  |  |
| χ^2^ (df) | 112.99 (2) | |
| RMSEA [90% CI] | .072 [.061, .084] | |
| CFI | .991 | |
| TLI | .974 | |
| α = .74 |  | |
| *Note.* Higher scores = higher online communication. Fit when email included: χ^2^ (5) = 112.79, *p* < .001; RMSEA = .045 [.038, .052]; CFI = .992; TLI = .983 however, α = .70 and λ = .19. | | |

| Poor Health | | |
| --- | --- | --- |
| **Items** | **λ** | **h^2^** |
| Headache | .77 | .59 |
| Stomach-ache | .72 | .52 |
| Back ache | .61 | .37 |
| Difficulties in sleeping | .57 | .33 |
| **Model fit** |  |  |
| χ^2^ (df) | 17.37 (2) | |
| RMSEA [90% CI] | .027 [.016, .039] | |
| CFI | .998 | |
| TLI | .995 | |
| α = .68 |  | |
| *Note.* Higher scores = worse health. α = .66 without sleep item | | |

| Teacher Support | | |
| --- | --- | --- |
| **Items** | **λ** | **h^2^** |
| Teacher accepts | .82 | .67 |
| Teacher cares | .92 | .85 |
| Feel trust in teacher | .87 | .75 |
| **Model fit** | Saturated | |
| α = .87 |  | |
| *Note.* Higher scores = higher support |  | |

| Classmate Support | | |
| --- | --- | --- |
| **Items** | **λ** | **h^2^** |
| Students being together | .73 | .53 |
| Students kind and helpful | .89 | .79 |
| Students accept me | .77 | .60 |
| **Model fit** | Saturated | |
| α = .79 |  | |
| *Note.* Higher scores = higher support |  | |

| Offline (Face-to-Face) Contact with Friends | | |
| --- | --- | --- |
| **Items** | **λ** | **h^2^** |
| Easy to talk to best friend | .91 | .83 |
| Talk to friends of same sex | .85 | .72 |
| Talk to friends of opposite sex | .57 | .32 |
| **Model fit** | Saturated | |
| α = .73 |  | |
| *Note.* Higher scores = more offline contact |  | |

| Loneliness | | |
| --- | --- | --- |
| **Items** | **λ** | **h^2^** |
| Feeling close to others | .50 | .25 |
| Last week: felt lonely | .78 | .61 |
| Feeling left out | .75 | .56 |
| **Model fit** | Saturated | |
| α = .68 |  | |
| *Note.* Higher scores = more frequent loneliness. | | |

**Measurement model (i.e., all latent factors together):**

Fit: χ^2^ (155) =2311.85, *p* < .001; RMSEA = .036 [.035, .037]; CFI = .974; TLI = .968.
